# Physiological and Metabolomic Responses of ‘Bluegold’ Blueberry to Infection by an Isolate Preliminarily Identified as *Diaporthe eres*

**DOI:** 10.3390/plants15142172

**Published:** 2026-07-15

**Authors:** Yuanzhen Wang, Suixin Cong, Jiaming Ju, Ruixue Guo, Xuedong Tang, Jianxin Li

**Affiliations:** Department of Horticulture, Jilin Agricultural University, Changchun 130118, China; 18803921879@163.com (Y.W.); 18143037268@163.com (S.C.); jjm827167460@163.com (J.J.)

**Keywords:** blueberry, *Diaporthe* isolate, oxidative stress, phenylpropanoid biosynthesis, flavonoids, metabolomics

## Abstract

Canker is a destructive fungal disease that adversely affects plant growth, triggering leaf drop, branch dieback and even plant death. For the blueberry industry, fungal canker reduces blueberry yield and quality. In this study, diseased branches of the ‘Bluegold’ blueberry cultivar collected from open-field plantations in Jilin Province, China, were used to isolate and identify the pathogen, characterize its biological properties, and investigate the physiological and metabolomic responses of blueberry shoots to pathogen infection. Based on morphological characteristics and internal transcribed spacer sequence analysis, the pathogen was preliminarily identified as *Diaporthe eres*. Biological characterization showed that the optimal temperature and pH for mycelial growth of the isolate were 25 °C and 6, respectively, while light conditions had no significant effect on fungal growth. Pathogenicity results demonstrated that infection by the isolate significantly induced the accumulation of superoxide anions (O_2_^.−^) and hydrogen peroxide (H_2_O_2_) in blueberry new shoots, which led to lipid peroxidation and subsequently resulted in significant increases in malondialdehyde content and relative electrical conductivity. Concurrently, the plant’s antioxidant enzyme system was disrupted, and the activities of peroxidase and superoxide dismutase remained significantly elevated throughout the infection period, while catalase activity exhibited an initial increase followed by a gradual decline. Metabolomic analysis identified 541 differentially accumulated metabolites, with key metabolites including ferulic acid, quercetin and kaempferol showing marked abundance changes. These were primarily enriched in pathways closely associated with plant resistance, such as phenylpropanoid biosynthesis and flavonoid biosynthesis. Combined analysis of physiological and metabolomic data showed that reactive oxygen species accumulation and antioxidant enzyme responses were accompanied by significant enrichment of phenylpropanoid and flavonoid metabolism, indicating coordinated antioxidant regulation and secondary metabolic reprogramming in blueberry shoots following pathogen infection. Overall, this study preliminarily identified a *D. eres* isolate associated with blueberry canker in Jilin Province, and systematically characterized the physiological and metabolic responses of blueberry to pathogen infection, providing a theoretical basis for understanding blueberry—*Diaporthe* interactions and for developing effective disease management strategies.

## 1. Introduction

Blueberries, belonging to the genus *Vaccinium* in the family Ericaceae, are perennial deciduous shrubs known for their rich antioxidants. They are reputed for their ability to combat memory decline, prevent cardiovascular and cerebrovascular diseases, and enhance eyesight, earning them the title of ‘King of Berries’ [1]. Owing to the unique climatic conditions and large diurnal temperature fluctuations in Northeast China, blueberries cultivated in this region generally exhibit higher sugar and anthocyanin contents. However, as the area under cultivation continues to expand and the duration of cultivation increases, the incidence of various diseases has become increasingly severe, significantly limiting blueberry yield and quality [2]. In particular, blueberry canker has occurred frequently in recent years in the northeastern production areas of China. During a survey of a blueberry orchard in Jilin, China (42°14′ N, 127°35′ E), it was found that canker was present in many varieties, with the ‘Bluegold’ variety being the most severely affected. The incidence rate of disease on its branches and the rate of branch dieback were both significantly higher than those of other major cultivated varieties, making it one of the most severely affected varieties in the region.

Fungi of the genus *Diaporthe* are plant pathogens with significant economic impact, characterized by a broad host range, high virulence, and difficulty in control. Fungi of this genus can infect a wide variety of fruit trees and crops, including blueberries, grapes, soybeans, and peanuts [3,4]. Different *Diaporthe* species can cause a variety of serious diseases. *Diaporthe ampelina* is the primary pathogen responsible for Phomopsis cane and leaf spot in grapes, causing shoot necrosis, leaf spots, and trunk diseases, thereby reducing grape yield and fruit quality; in addition, species such as *D. eres*, *D. foeniculina*, and *D. rudis* are also associated with Phomopsis cane and leaf spot and canker diseases in grapes [5]. *D. caulivora* and *D. aspalathi* are the primary pathogens causing soybean stem canker, while *D. sojae* is the primary pathogen causing pod and stem blight; *D. longicolla* primarily causes *Diaporthe* seed decay. These diseases can lead to reduced seed quality, lower germination rates, and premature plant senescence; in severe cases, they can even result in significant yield losses [6]. *Diaporthe* species are among the major pathogens associated with blueberry canker; currently known species capable of causing blueberry diseases include *Diaporthe amygdali* [7], *Diaporthe phaseolorum* [8], *Diaporthe australafricana* [8], and *Diaporthe vaccinii* [9]. This disease primarily affects blueberry shoots; the pathogen invades the shoots, causing them to wither and, in severe cases, die, resulting in a significant decline in fruit quality and yield [10,11,12].

In recent years, as research on plant–pathogen interactions has deepened, it has been discovered that pathogen infection not only causes damage to tissue structure but also induces complex physiological and biochemical responses and metabolic reprogramming in the host plant. Previous studies on blueberry diseases have shown that pathogen infection can activate antioxidant defense systems and phenylpropanoid metabolism, resulting in enhanced accumulation of phenolic compounds and flavonoids associated with disease resistance [13]. Similar physiological and metabolic responses have also been reported in other plant–pathogen interaction systems, including bacterial canker of kiwifruit, grapevine trunk diseases, pumpkin powdery mildew, and cotton Verticillium wilt [14,15,16,17,18]. Moreover, numerous studies have shown that reactive oxygen species (ROS), including superoxide anions (O_2_^.−^) and hydrogen peroxide (H_2_O_2_), rapidly accumulate following pathogen infection. In plants, this early ROS burst is primarily generated by plasma membrane-localized respiratory burst oxidase homologs (RBOHs), which are rapidly activated after pathogen recognition and play a central role in initiating immune signaling [19]. However, excessive or prolonged ROS accumulation may disrupt cellular redox homeostasis and ultimately lead to oxidative damage. Following infection by pathogens in various fruit trees and crops, the accumulation of ROS, membrane lipid peroxidation, and the activation of antioxidant enzymes have been observed [14,15,16,17,18]. Increasing evidence indicates that ROS function not only as major contributors to oxidative damage during pathogen infection but also as key signaling molecules regulating plant defense responses. Pathogen-induced ROS accumulation can activate a series of defense-related signaling pathways and further influence the reprogramming of plant secondary metabolism [20]. Among these, the phenylpropanoid metabolic pathway and its downstream flavonoid biosynthesis pathway are considered key metabolic networks in plants’ response to biotic stress [21]. Relevant studies have shown that the phenolic acids and flavonoids produced by these pathways not only participate in ROS scavenging and the maintenance of redox homeostasis but also enhance plant disease resistance by promoting cell wall reinforcement, regulating immune signaling, and inhibiting pathogen growth [22]. Therefore, the accumulation of ROS may serve as an upstream signal linking the activation of the antioxidant defense system to metabolic reprogramming following pathogen infection, jointly contributing to the plant’s response to pathogen invasion.

Although several studies have reported the occurrence, taxonomy, and pathogenic characteristics of *Diaporthe* species associated with blueberry diseases, recent research has mainly focused on pathogen identification and disease symptoms. Very little information is currently available regarding the dynamic physiological responses of blueberries during infection by *Diaporthe* pathogens, particularly the temporal changes in ROS levels, antioxidant enzyme activity, and membrane lipid peroxidation as the disease progresses. Moreover, although metabolomic approaches have been increasingly applied to the study of plant–pathogen interactions, the metabolic reprogramming induced by *Diaporthe* infection in blueberries remains unexplored. Consequently, this study focused on the canker disease affecting the ‘Bluegold’ blueberry cultivar in Jilin Province, China. The objectives were to isolate and preliminarily identify the pathogen associated with blueberry canker, observe its biological properties under different environmental conditions, investigate the physiological responses of blueberry shoots to pathogen infection by monitoring ROS accumulation, membrane damage, and antioxidant enzyme activities, and further elucidate the metabolic reprogramming underlying the host defense response using untargeted metabolomics. By integrating pathogen characterization with physiological and metabolomic analyses, this study provides new insights into the defense responses of blueberry to canker-associated *Diaporthe* infection and establishes a theoretical foundation for future functional validation of defense-related metabolites and the development of effective disease management strategies.

## 2. Results

### 2.1. Survey of Diseases in Orchards

**Figure 1:**
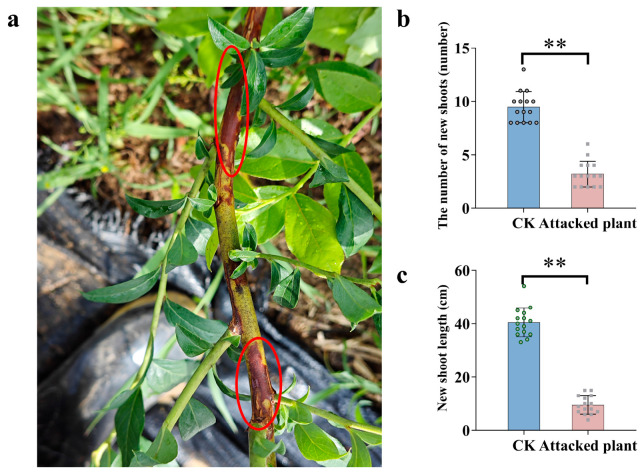
Effects of blueberry canker on shoot growth under field conditions. (**a**) Symptoms of canker on blueberry branches, the area circled in red is the site of the lesion; (**b**) Average number of new shoots per main stem in healthy (CK) and diseased plants; (**c**) Average length of new shoots in CK and diseased plants. Values are means ± SD (*n* = 15). Significant differences between healthy (CK) and diseased plants were determined using Student’s *t*-test. Asterisks indicate significant differences (** *p* < 0.01).

To investigate the impact of branch canker disease in blueberry orchards in Jilin, China, under open-field cultivation conditions, a disease survey and statistical analysis were conducted during the first growth peak of the blueberry new shoots. Approximately 50% of the surveyed blueberry plants exhibited typical canker symptoms, indicating that the disease was widespread in the orchard. Among the surveyed cultivars, ‘Bluegold’ showed the highest incidence of branch canker and was therefore selected for subsequent pathogen isolation and physiological analyses. Infected plants exhibited distinct reddish-brown branch lesions, which markedly inhibited shoot growth (Figure 1a). Survey data indicated that healthy plants (CK) produced an average of 10 new shoots per main stem, with an average new shoot length of 40 cm. In contrast, diseased plants produced an average number of only four new shoots per main stem (Figure 1b), and the average shoot length was merely 10 cm (Figure 1c). In summary, both the number and length of new shoots on healthy plants were significantly higher than those on diseased plants (*p* < 0.01), indicating that blueberry canker significantly inhibited the emergence and growth of new shoots.

### 2.2. Isolation and Identification of the Pathogen

**Figure 2:**
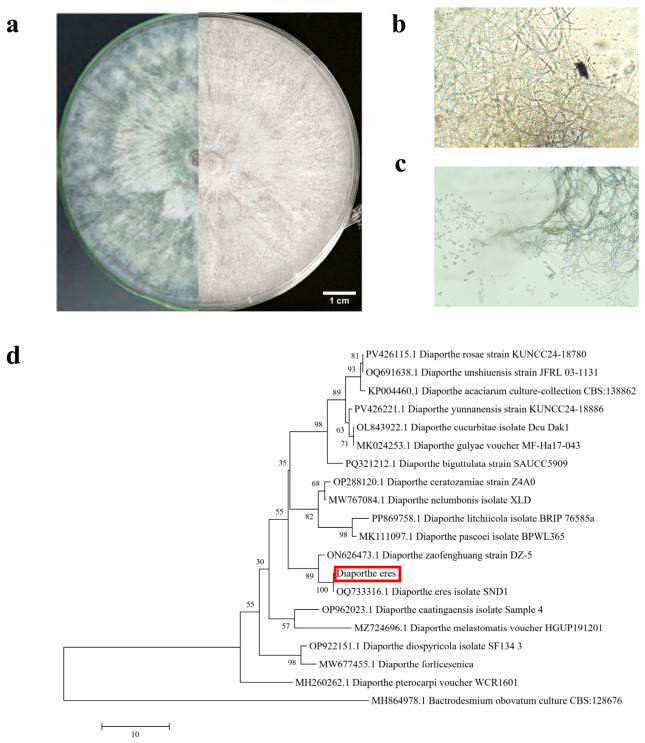
Preliminary identification of the *Diaporthe* isolate. (**a**) Comparison chart of the isolated strain and *D. eres* (**b**) structure of conidiophores; (**c**) septation of hyphae and conidia morphology; and (**d**) phylogenetic tree analysis of *Diaporthe eres* pathogens.

To determine the cause of blueberry shoot canker, pathogen isolation and identification were carried out. Pathogens were isolated and purified using PDA medium, and internal transcribed spacer (ITS) sequencing was performed on the isolated pathogens. A total of four fungal genera were isolated and identified, including *Diaporthe*, *Epicoccum*, *Acremonium*, and *Simplicillium*, among which *Diaporthe* exhibited pathogenic characteristics most similar to those of blueberry shoot canker (Figure S1). The phylogenetic tree constructed using the neighbor-joining method indicated that when Bactrodesmium obovatum culture CBS:128676 (MH864978.1) was used as an outgroup, this isolate clustered closely with the reference strain of *Diaporthe eres* (Figure 2d). Based on its morphological characteristics and ITS sequence analysis, the isolate was preliminarily identified as *D. eres*. However, because additional phylogenetically informative loci (TEF1-α, TUB2, HIS3, and CAL) were not included in this study, the species assignment should be regarded as preliminary and requires further confirmation by multi-locus phylogenetic analysis.

Observation of *D. eres* culture plates after 7 days of incubation revealed that the hyphae had completely covered the dish, presenting an overall hair-like appearance. During cultivation, the color changed from white to greyish-white and then to light grey, and the marginal areas were irregular, relatively sparse, flat, and lighter in color (Figure 2a). After 25 days of cultivation, the mycelium was colorless or light grey, slender, branched, and black bore, spherical to sub-spherical pycnidia and hyaline, and oval-to-fusiform conidia were observed (Figure 2b,c).

### 2.3. The Effect of Environmental Factors on the Growth of the Isolate

**Figure 3:**
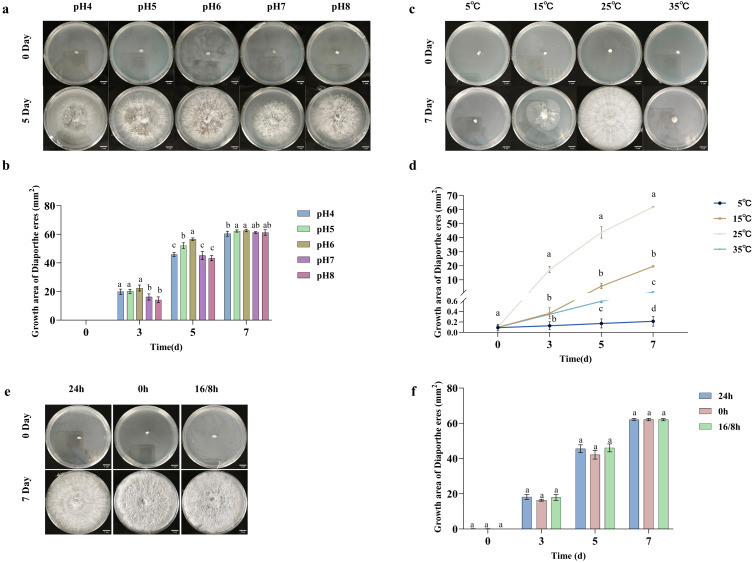
Growth of the *Diaporthe* isolate preliminarily identified as *D. eres* under different environmental conditions. (**a**) Colony morphology and (**b**) growth area at different pH levels. (**c**) Colony morphology and (**d**) growth area at different cultivation temperatures. (**e**) Colony morphology and (**f**) growth area under different light conditions, 24 h indicates full light, 0 h indicates complete darkness, and 16/8 h light–dark cycle indicates 16 h of light and 8 h of darkness. All physiological measurements were obtained from three biological replicates, each with three technical replicates. Significance of differences was analyzed using analysis of ANOVA and Tukey HSD test; different lowercase letters indicate significant differences among treatment groups (*p* < 0.05).

The effects of pH, temperature, and light on the mycelial growth of the isolate were evaluated. Under conditions where temperature and light were kept constant, by the 5th day of cultivation, the colony area at pH 6 was significantly larger than that of the other pH gradient groups (Figure 3a,b). Temperatures were set between 5 and 35 °C, and the isolate strain grew most rapidly at 25 °C, whereas temperatures of 5 °C and 35 °C significantly inhibited growth (Figure 3c,d). Furthermore, different photoperiods—including darkness, full daylight, and 16 h of light—did not result in significant differences in morphogenesis or growth rate (Figure 3e,f). These results indicate that the isolate exhibits strong environmental adaptability, capable of growing under both slightly acidic and slightly alkaline pH conditions. Low and high temperatures inhibit its growth rate but do not inactivate the fungus, while light exposure does not affect its growth.

### 2.4. Virulence Testing of D. eres

**Figure 4:**
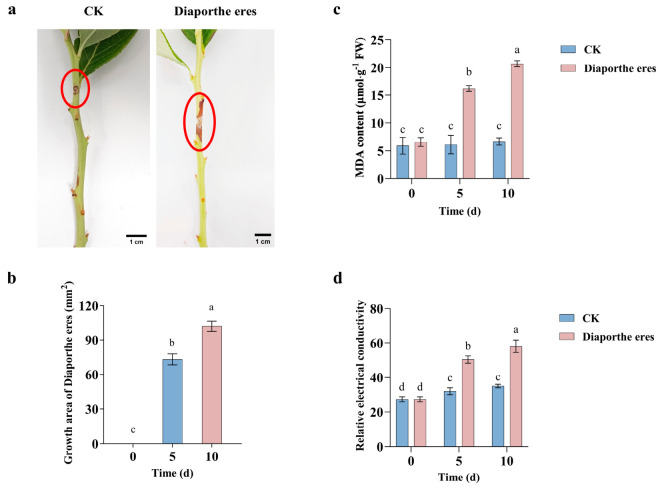
Pathogenicity of the isolate on blueberry shoots. (**a**) Symptoms and phenotypic characteristics of lesions, the area circled in red is the site of the lesion; (**b**) infection area; (**c**) MDA content; and (**d**) relative electrical conductivity 10 days after inoculation of healthy blueberry new shoots with the pathogen *Diaporthe eres*. All physiological measurements were obtained from three biological replicates, each with three technical replicates. Significance of differences was analyzed using analysis of ANOVA and Tukey HSD test; different lowercase letters indicate significant differences among treatment groups (*p* < 0.05).

In order to verify the pathogenicity of the isolate on blueberry shoots, the pathogen was inoculated onto healthy blueberry shoots. The inoculation wounds in CK gradually healed 10 days after inoculation. In contrast, 5 days after inoculation with *D. eres*, the shoots exhibited distinct signs of disease, with reddish-brown lesions appearing over an area of 73 mm^2^. As the post-inoculation period progressed, the lesions continued to spread, and 10 days after inoculation, the lesion area increased by 40% compared to day 5, reaching 102 mm^2^, with a clear demarcation between healthy and diseased tissue (Figure 4a,b). The pathogen was re-isolated from randomly selected symptomatic branches, and the ITS sequences of the reselected strains matched those of the inoculated strains.

### 2.5. Physiological Responses of Blueberry Shoots to Infection by the Isolate

**Figure 5:**
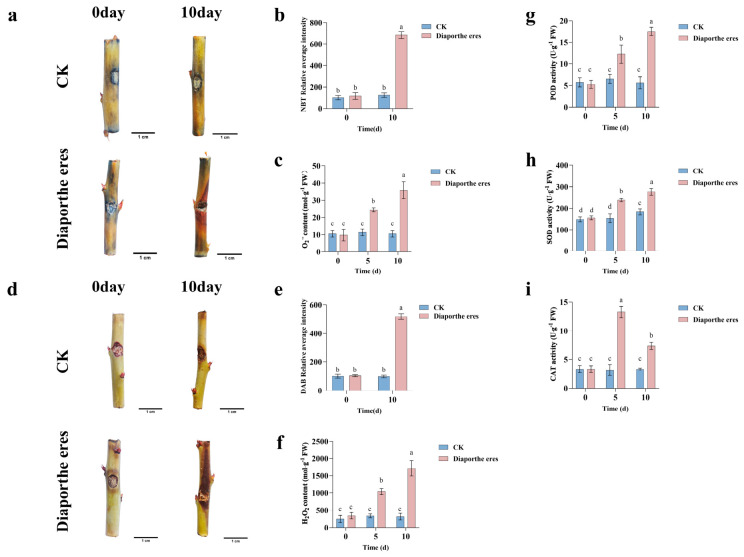
The effects of the isolate on the antioxidant defense system of blueberries. (**a**) NBT staining; (**b**) NBT-stained intensity; (**c**) O_2_^.−^ generation rate; (**d**) DAB staining; (**e**) DAB-stained intensity; (**f**) H_2_O_2_ content; (**g**) POD activity; (**h**) SOD activity; and (**i**) CAT activity of blueberry new shoots. All physiological measurements were obtained from three biological replicates, each with three technical replicates. Significance of differences was analyzed using analysis of ANOVA and Tukey HSD test; different lowercase letters indicate significant differences among treatment groups (*p* < 0.05).

To investigate the effects of the isolate on the antioxidant defense system of blueberry shoots, the levels of O_2_^.−^ and H_2_O_2_ were measured. The results showed that, as the infection progressed, the levels of O_2_^.−^ and H_2_O_2_ in the shoots were significantly higher than those in CK, increasing by 237.85% and 441.14%, respectively, by day 10. Consistent with these quantitative measurements, ImageJ 1.54p-based quantification of histochemical staining revealed that the staining intensities of 3,3′-diaminobenzidine (DAB) and nitroblue tetrazolium (NBT) remained relatively unchanged in the control, whereas they increased significantly in infected shoots, reaching 491.31% and 401.64% of the control levels, respectively, at 10 days after inoculation (Figure 5a–f), indicating that the pathogen *D. eres* caused excessive accumulation of ROS in the plants.

Peroxidase (POD), superoxide dismutase (SOD), and catalase (CAT) are key antioxidant enzymes in the antioxidant system, and their activities reflect the extent of damage caused by external factors. POD and SOD activities increased with the passage of time following inoculation, and by day 10, they had increased by 211.55% and 50.62%, respectively, compared to healthy plants (Figure 5g,h). CAT activity initially rose and then declined, peaking on day 5 and falling by 44.39% by day 10 (Figure 5i).

Toluidine blue staining results indicated that cells in CK remained unstained, whereas cross-sections of branches inoculated with *D. eres* at 10 days exhibited a distinct blue color. This suggests that *D. eres* inoculation caused damage to the cell membranes of blueberry branches, leading to cell death (Figure S2). Furthermore, compared with the control, as the inoculation time increased, the malondialdehyde (MDA) content in the diseased shoots increased 1.65-fold and 2.11-fold on days 5 and 10, respectively, and similarly, the relative electrical conductivity of the diseased shoots increased 0.57-fold and 0.66-fold, respectively (Figure 4c,d).

### 2.6. Principal Component Analysis

To elucidate the metabolic responses of blueberry shoots following infection by the isolate, untargeted metabolomic analysis was performed 10 days after inoculation. According to the PCA analysis, the three replicates of the same treatment clustered closely together, indicating that the experimental data were stable and reproducible. There were marked metabolic differences between the treatment groups, indicating that pathogen infection significantly altered the metabolic profile of blueberry shoots (Figure S3).

To further validate the metabolic differences between the control group and the infected group, supervised orthogonal partial least squares discriminant analysis (OPLS-DA) was performed. Samples from the CK and D groups were clearly separated, indicating that the two groups exhibited distinct metabolic profiles. Furthermore, the corresponding permutation tests confirmed that all OPLS-DA models were robust, and no signs of overfitting were observed, supporting the reliability of subsequent differential metabolite screening (Figure S4). These results indicate that infection by the isolate substantially reshaped the global metabolic profile of blueberry shoots, providing a foundation for identifying defense-related metabolic pathways.

### 2.7. Analysis of Differentially Accumulation Metabolites

**Figure 6:**
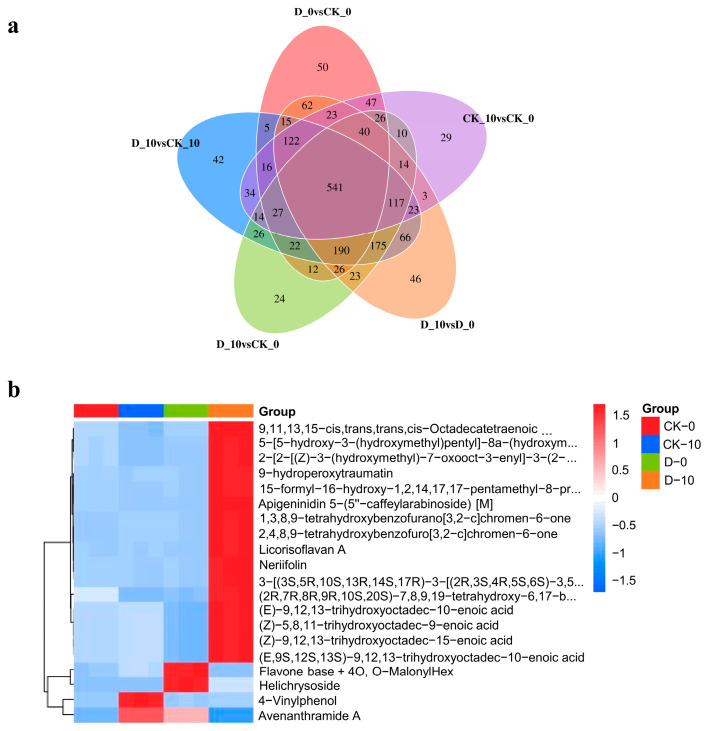
(**a**) Venn diagram of DAMs among the four treatment groups (VIP ≥ 1, *p*-value < 0.05, |log_2_FC| ≥ 1). (**b**) Hierarchical clustering heatmap comparing the four groups CK-0, CK-10, D-0, and D-10. The colored blocks at different positions represent the relative expression levels of the corresponding metabolites; red indicates high expression of the substance in that group, while blue indicates low expression. CK-0 and CK-10 designate blueberry new shoots cultured for 0 and 10 days, respectively, without *Diaporthe eres* inoculation; D-0 and D-10 designate blueberry new shoots cultured for 0 and 10 days, respectively, post-inoculation with *Diaporthe eres* (*p*-value < 0.01).

Venn diagrams were constructed to compare the differentially accumulated metabolites (DAMs) identified among the treatment groups. Based on the screening criteria (Variable importance in projection (VIP) ≥ 1, *p*-value < 0.05, |log_2_FC| ≥ 1), a total of 541 common DAMs were identified across the five treatment groups. These metabolites were associated with amino acid metabolism, secondary metabolism, and other metabolic processes, with many involved in signaling and defense regulation. This result indicated that 541 DAMs were shared among different treatment groups and remained responsive throughout the infection process in blueberry shoots (Figure 6a). The persistence of these DAMs across treatment comparisons suggests that they represent core metabolic responses of blueberry to *D. eres* infection rather than transient metabolic fluctuations.

### 2.8. Hierarchical Cluster Analysis of Differential Accumulation Metabolites

The heatmap from the hierarchical cluster analysis showed that the relative expression levels of metabolites in treatment groups CK-0 and CK-10 were relatively similar. The relative expression levels of metabolites in treatment groups CK-0 and D-0 were relatively similar, whereas those in treatment groups D-0 and D-10 differed significantly. The data indicated that progressive metabolic reprogramming occurred following pathogen infection (Figure 6b and Figures S5–S7). Among them, ferulic acid, quercetin, kaempferol, and other metabolites related to phenylpropanoid biosynthesis, flavonoid biosynthesis, and antioxidant-related pathways were significantly enriched, indicating that these metabolic pathways may be involved in the defense response of blueberry to pathogen infection. These results suggest that pathogen-induced metabolic changes were mainly associated with activation of secondary metabolism rather than broad metabolic disruption.

### 2.9. K-Means Analysis

**Figure 7:**
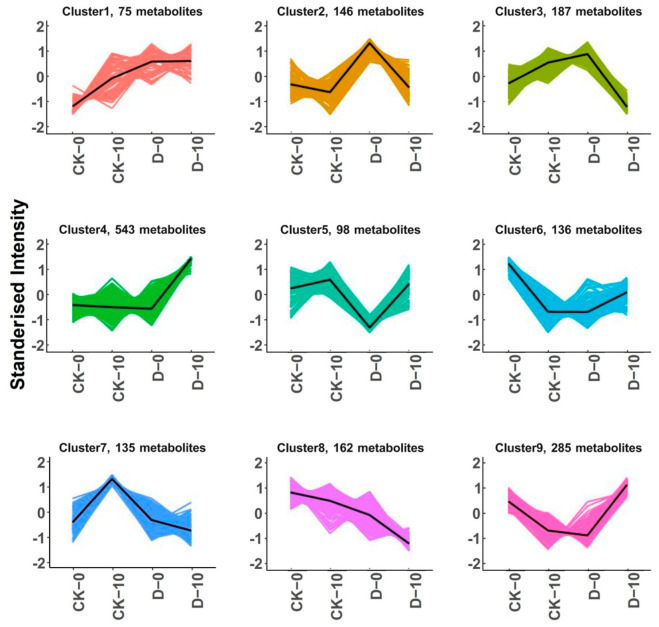
K-Means plots for all groups. Each curve illustrates the trend in the relative abundance of a specific class of metabolites across various groups. The *X*-axis denotes the experimental groups, while the *Y*-axis reflects the normalized abundance of metabolites. The numbers in parentheses adjacent to each cluster indicate the quantity of metabolites within that class.

To characterize dynamic metabolic changes during pathogen infection, the relative abundances of all identified DAMs were standardized using z-scores and subjected to K-means clustering analysis. Based on the trends in standardized abundance of metabolites across the four treatment conditions (CK-0, CK-10, D-0, and D-10), the DAMs were classified into nine major expression profiles. Some metabolite clusters showed progressive up-regulation after inoculation, indicating that defense-related metabolic pathways were activated under pathogen stress. On the contrary, other metabolic clusters were continuously down-regulated, indicating that primary metabolism may be inhibited during pathogen infection. Taken together, these results suggest coordinated activation of defense-related secondary metabolism together with suppression of selected primary metabolic processes during infection by the isolate (Figure 7).

### 2.10. KEGG Pathway Analysis

**Figure 8:**
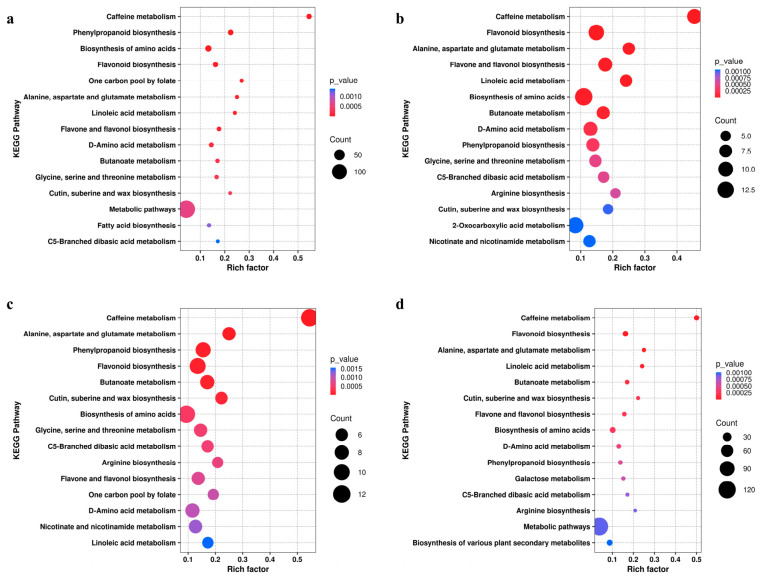
KEGG enrichment plots comparing DAMs across treatment groups. (**a**) CK-0 vs. CK-10 vs. D-0 vs. D-10; (**b**) D-10 vs. CK-0; (**c**) D-10 vs. CK-10; (**d**) D-10 vs. D-0. The *x*-axis represents the Rich Factor for each pathway, whilst the *y*-axis shows the names of the KEGG metabolic pathways. The size of the dots indicates the number of DAMs enriched in that pathway. The color indicates the magnitude of the *p*-value; the smaller the *p*-value, the more reddish the color, indicating a more significant level of enrichment.

To investigate the metabolic pathways associated with the identified DAMs, the screened metabolites were mapped to the KEGG database for pathway annotation and enrichment analysis. Based on the classification statistics, the DAMs screened from the four treatment groups were annotated across 20 metabolic pathways, covering 7 major functional categories (Figure 8).

KEGG enrichment analysis revealed that many DAMs were associated with pathways involved in the biosynthesis of secondary metabolites. Among these, phenylpropanoid biosynthesis, flavonoid biosynthesis, and flavone and flavonol biosynthesis were enriched across treatment comparisons. Within these pathways, several intermediate and downstream metabolites exhibited distinct accumulation patterns during infection. In particular, ferulic acid, a representative phenylpropanoid metabolite, and the flavonoids quercetin and kaempferol accumulated markedly in infected shoots compared with the corresponding controls. The coordinated accumulation of these metabolites suggests activation of the phenylpropanoid–flavonoid metabolic network during infection by the isolate, indicating that secondary metabolic reprogramming is closely associated with the blueberry defense response. These pathways therefore represent major metabolic processes associated with the response of blueberry to pathogen infection.

### 2.11. Integrated Analysis of Physiological Responses and Metabolic Reprogramming

**Figure 9:**
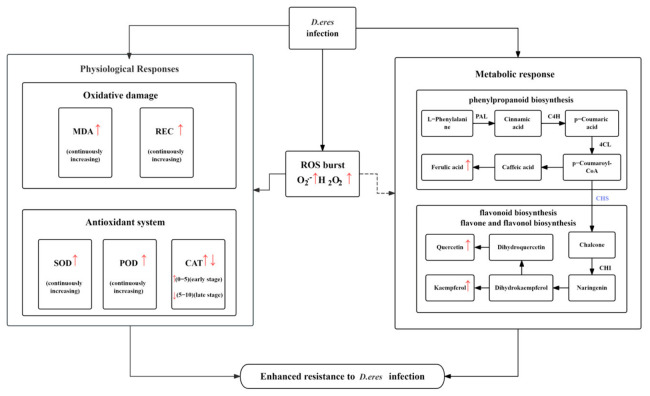
Response diagram of physiological and metabolic changes in blueberry shoots following infection by *D. eres*. Red upward arrows indicate increased accumulation or activity, whereas the red downward arrow indicates decreased activity during the infection process. Dashed arrows represent putative regulatory relationships inferred from the integration of physiological and metabolomic analyses rather than experimentally validated causal interactions. Purple enzyme abbreviations indicate representative enzymes involved in phenylpropanoid and flavonoid biosynthesis pathways.

Figure 9 summarizes the integrated physiological and metabolomic responses of blueberry shoots to pathogen infection. The results indicate that infection by the isolate triggered a significant accumulation of ROS, including O_2_^.−^ and H_2_O_2_, accompanied by increased MDA levels and relative electrical conductivity, suggesting that oxidative stress intensified and cell membranes were damaged following pathogen infection. Concurrently, antioxidant enzymes were activated to maintain redox homeostasis. Metabolomic analysis further showed increased accumulation of metabolites associated with phenylpropanoid biosynthesis, flavonoid biosynthesis, and flavone and flavonol biosynthesis, with representative metabolites including ferulic acid, quercetin, and kaempferol exhibiting markedly higher abundance in infected shoots than in the corresponding controls. Taken together, these results suggest that infection by the isolate induced oxidative stress in blueberry shoots and is accompanied by activation of the antioxidant defense system and extensive secondary metabolic reprogramming.

## 3. Discussion

Blueberry stem canker is an important fungal disease that severely affects plant growth and productivity. In the present study, the pathogen associated with blueberry stem canker in Jilin Province was preliminarily identified as a *Diaporthe eres* isolate based on morphological characteristics and ITS sequence analysis. Physiological and metabolomic analyses further revealed that infection by the isolate induced substantial oxidative stress, activated antioxidant defense responses, and triggered extensive metabolic reprogramming in blueberry shoots. These findings provide new insights into the physiological and metabolic mechanisms underlying blueberry responses to *D. eres* infection.

### 3.1. Pathogen Identification and Pathogenic Characteristics of D. eres

In the present study, the isolate was preliminarily identified as a *Diaporthe eres* isolate based on morphological characteristics and ITS sequence analysis. Although ITS sequencing is widely used for the preliminary identification of *Diaporthe* species, multilocus phylogenetic analyses are generally recommended to improve species resolution within this taxonomically complex genus [23,24]. Previous studies have demonstrated that loci such as TEF1-α, TUB2, HIS3, and CAL provide higher discriminatory power for closely related *Diaporthe* species [23,24]. Accordingly, the identification presented here should be regarded as preliminary, and future multilocus analyses will further refine the taxonomic placement of this isolate. Nevertheless, the isolate consistently exhibited colony morphology, pathogenicity, and ITS phylogenetic characteristics that were consistent with those reported for *D. eres*, thereby supporting its preliminary identification [23,25].

This study found that the isolate primarily infected blueberry shoots (Figure 4), causing blueberry stem canker, whereas other studies have reported infection mainly in stem buds, leading to bud blight [26]. These differences in infection sites may be attributed to two factors: firstly, variations in the resistance of different blueberry cultivars to the pathogen [27], as research has shown that there are significant differences in the susceptibility of different blueberry cultivars to fungi of the genus *Diaporthe*; and secondly, the fact that *D. eres* exhibits high intraspecific variability between different isolates [28], and certain strains (such as CBS 160.32) have low virulence, while others (such as CAA829) exhibit high virulence. These observations suggest that disease development in blueberries may be influenced by both the genetic diversity of *D. eres* and host cultivar susceptibility.

### 3.2. Oxidative Stress and Redox Regulation During D. eres Infection

During pathogen infection, the host generally rapidly produces a large amount of ROS, such as O_2_^.−^ and H_2_O_2_, which act as signaling molecules to initiate a series of defense responses [29,30]. The marked accumulation of O_2_^.−^ and H_2_O_2_ observed following *D. eres* infection indicates that an oxidative burst constitutes an early defense response of blueberry to pathogen invasion (Figure 5c,f). This response may be attributed to the secretion of cell wall-degrading enzymes, such as pectinase and laccase, which disrupt the structural integrity of blueberry tissues, release damage-associated molecular patterns (DAMPs), and subsequently trigger NADPH oxidase-mediated ROS production, leading to tissue necrosis [31,32]. The sustained increase in SOD and POD activities further suggests that blueberry continuously activated its antioxidant defense system to counteract excessive ROS accumulation (Figure 5g,h). Similar dynamic changes in antioxidant enzyme activities have also been reported during *D. eres* infection of Hongyang kiwifruit, indicating that activation of enzymatic ROS-scavenging systems may represent a conserved defense response to *Diaporthe* infection [33]. The concurrent increase in MDA content and relative electrical conductivity indicates that prolonged ROS accumulation exceeded the antioxidant buffering capacity of the host and resulted in membrane lipid peroxidation and membrane damage. Similar oxidative damage has also been reported in soybean infected by *D. eres* and in wheat infected by Bipolaris sorokiniana, suggesting that excessive ROS accumulation is a common physiological consequence of fungal infection [34,35]. Because *D. eres* primarily colonizes perennial woody tissues, prolonged membrane damage may directly contribute to vascular dysfunction and subsequent shoot dieback in blueberry. These observations suggest that the level of ROS induced by *D. eres* in blueberry exceeded that required for signaling alone and was sufficient to cause pronounced oxidative injury, thereby necessitating the activation of antioxidant defense mechanisms. Recent studies have demonstrated that ROS do not function solely as cytotoxic molecules but also act as key signaling intermediates during plant–pathogen interactions. In particular, ROS and reactive nitrogen species (RNS) are increasingly recognized as coordinated regulators of redox signaling networks that integrate immune activation, antioxidant metabolism, hormonal signaling, and stress adaptation [36]. The balance between ROS production and scavenging is critical for determining the outcome of plant–pathogen interactions. Moderate ROS accumulation promotes defense signaling, whereas excessive ROS causes oxidative damage. Therefore, the dynamic changes in ROS and antioxidant enzyme activities observed in this study suggest that blueberry initially activates antioxidant defenses to maintain redox homeostasis, but prolonged pathogen infection ultimately disrupts this balance, leading to oxidative injury and disease progression.

In this study, CAT activity exhibited a trend of initially increasing and then decreasing (Figure 5i). During the early stages of infection, plants may have rapidly cleared excess H_2_O_2_ by activating their antioxidant defense system, thereby alleviating the oxidative stress induced by the pathogen. However, as the disease progressed, CAT activity declined significantly, indicating a gradual imbalance in the host’s redox homeostasis. This may be attributed to two factors. On the one hand, prolonged pathogen infection leads to excessive ROS accumulation, which not only causes membrane lipid peroxidation but may also oxidatively inactivate antioxidant enzymes, thereby reducing CAT activity [37,38]. In addition, sustained oxidative stress can alter the expression of CAT and other ROS-scavenging genes through redox-sensitive signaling pathways, further weakening the antioxidant capacity of host plants [39,40]. On the other hand, the pathogen may actively interfere with the host’s antioxidant defense system to promote colonization and expansion. Previous studies have shown that some plant pathogens can suppress host antioxidant enzyme activity by secreting effector proteins or regulating the expression of host redox-related genes. Whether *D. eres* employs similar mechanisms to interfere with host CAT activity remains unclear and deserves further investigation. During the infection of tobacco by *Phytophthora nicotianae* [41], the pathogen upregulates its own peroxisomal CAT activity while simultaneously suppressing the expression of host CAT-related genes, thereby impairing the host’s ability to clear H_2_O_2_ and promoting disease progression. In addition, previous studies have suggested that CAT is not only a key antioxidant enzyme responsible for the detoxification of H_2_O_2_ in plants but also plays an important role in immune regulation during plant–microbe interactions. A decline in CAT activity is often closely associated with the breakdown of host defense systems [42]. Therefore, the decline in CAT activity observed in this study may not only result from the continuous depletion of the host’s antioxidant system but may also be associated with the pathogen’s active interference with the host’s redox regulatory network. This suggests that during the late stages of disease development, plants may gradually transition from an early active defense phase to a state of oxidative defense imbalance and susceptibility, with the decline in CAT activity serving as a key physiological indicator of this transition.

### 3.3. The Functional Significance of Metabolic Reprogramming and DAMs Accumulation During D. eres Infection

Metabolomic analysis in this study revealed that following infection by pathogenic fungi, the levels of DAMs such as quercetin, kaempferol, and ferulic acid were significantly elevated in blueberry plants. These DAMs were primarily associated with pathways such as phenylpropanoid biosynthesis, flavonoid biosynthesis, and flavone and flavonol biosynthesis (Figure 6 and Figure 8). Although comparable metabolomic studies focusing on *D. eres* infection in blueberry remain limited, similar activation of these pathways has been reported in several fruit tree–pathogen interaction systems, suggesting that secondary metabolic reprogramming represents a conserved defense strategy against fungal pathogens. In apples and *Venturia inaequalis* [43,44], the levels of phenylpropanoid metabolites such as ferulic acid, quercetin, and kaempferol in apple leaves increased significantly following inoculation, while SOD and POD activities were also enhanced. Studies have shown that following infection with blueberry gray mold [13], the activity of key enzymes in the phenylpropanoid pathway, such as PAL, C4H and 4CL, increased significantly, accompanied by an accumulation of total phenolic and flavonoid content. In the response of the medicinal plant Coptis to root rot [45], the levels of kaempferol and quercetin derivatives increased significantly with increasing disease severity, while genes in the flavonoid biosynthetic pathway were progressively up-regulated. These studies indicate that phenylpropanoid biosynthesis and its downstream flavonoid biosynthesis are key defensive metabolic processes in plants’ response to pathogen infection. 

The enrichment of phenylpropanoid biosynthesis, flavonoid biosynthesis, and flavone and flavonol biosynthesis observed in the present study further supports the activation of inducible defense metabolism following the isolate infection. Phenylpropanoid metabolism provides precursors for lignin biosynthesis and various phenolic compounds, which contribute to cell wall reinforcement and restrict pathogen spread within host tissues [46]. Meanwhile, flavonoid-related pathways generate a wide range of antioxidant and defense-associated metabolites that can scavenge excessive ROS, maintain redox homeostasis, and participate in immune signaling [47]. Together, these pathways likely function cooperatively to enhance the ability of blueberry shoots to tolerate oxidative stress and limit pathogen colonization.

It is worth noting that the accumulation of quercetin, kaempferol, and ferulic acid may not only reflect the activation of the host defense response but may also directly contribute to the development of plant disease resistance. Previous studies have shown that quercetin and kaempferol possess strong antioxidant properties, effectively scavenging the excessive ROS generated during pathogen infection, thereby reducing oxidative damage and maintaining cellular redox homeostasis [48,49]. In addition to their antioxidant functions, flavonoids also play a role in regulating plant immune signaling pathways and can enhance the host’s disease resistance by modulating the expression of defense-related genes [50,51]. Meanwhile, ferulic acid, as one of the key end products of the phenylpropanoid metabolic pathway, promotes the deposition of lignin and cell wall phenolic polymers, thereby enhancing the mechanical strength of the cell wall and limiting the spread of pathogens within the tissue [52,53]. Previous studies have shown that, during pathogen infection, activation of the phenylpropanoid pathway and lignin deposition are often concentrated in host cells surrounding infection sites, leading to the local accumulation of phenolic compounds that reinforce cell walls and restrict pathogen colonization. In apple infected by Venturia inaequalis, enhanced phenylpropanoid metabolism and lignification were predominantly associated with infected tissues during the early stages of infection, while similar localized reinforcement of the cell wall has also been reported in grapevine trunk diseases [43,46]. Because metabolomic profiling in the present study was performed using whole infected shoots, it was not possible to distinguish between local metabolite biosynthesis and long-distance metabolite transport. Nevertheless, the substantial accumulation of ferulic acid, quercetin, and kaempferol observed in this study is consistent with localized defense activation in infected blueberry tissues. Moreover, increasing evidence suggests that quercetin, kaempferol, and ferulic acid can inhibit mycelial growth, spore germination, and biofilm formation in several phytopathogenic fungi [54]. These antifungal effects are believed to be associated with disrupting the integrity of the fungal cell membrane, inducing intracellular oxidative stress, and interfering with fungal energy metabolism, ultimately inhibiting fungal growth and development [54]. Therefore, the accumulation of these metabolites observed in the present study may represent not only biomarkers of host defense activation but also potential chemical defenses involved in restricting *D. eres* infection. However, because no in vitro antifungal assays or spore germination tests were performed in the present study, it remains unclear whether quercetin, kaempferol, and ferulic acid directly inhibit the mycelial growth or spore germination of *D. eres*. Future studies should further evaluate the antifungal activity and underlying mechanisms of these metabolites through in vitro antifungal assays, mycelial growth analyses, and metabolite complementation experiments.

### 3.4. Synergistic Defense Mechanisms Involving the Antioxidant System and Metabolic Reprogramming

Infection by *D. eres* induced pronounced physiological and metabolic changes in blueberry shoots. ROS accumulation was accompanied by dynamic changes in antioxidant enzyme activities, while metabolomic analysis revealed significant enrichment of phenylpropanoid biosynthesis, flavonoid biosynthesis, and flavone and flavonol biosynthesis, together with increased accumulation of quercetin, kaempferol, and ferulic acid (Figure 9). These changes suggest that oxidative stress responses and secondary metabolic reprogramming occur simultaneously during pathogen infection. Previous studies have shown that enhanced antioxidant enzyme activities are frequently associated with activation of phenylpropanoid metabolism and accumulation of phenolic and flavonoid compounds during disease resistance in blueberry and other plant species [55,56]. Furthermore, ROS generated after pathogen recognition can activate defense signaling pathways and promote the biosynthesis of phenylpropanoid- and flavonoid-derived metabolites involved in antioxidant protection and structural defense [57]. Although the present study did not perform correlation analyses between physiological traits and metabolite abundance, the consistent trends observed in both datasets suggest a close association between oxidative stress responses and metabolic reprogramming during *D. eres* infection. Accordingly, Figure 9 summarizes the physiological and metabolomic responses observed in this study and illustrates a hypothetical framework describing their potential relationships. Further experimental validation will be required to determine whether these relationships are causally linked. However, because hormone signaling pathways, particularly salicylic acid (SA) and jasmonic acid (JA), were not investigated in the present study, their contributions to ROS signaling and secondary metabolic reprogramming remain unclear. Further studies integrating hormone analyses with physiological and metabolomic approaches are needed to clarify these regulatory relationships.

Although this study revealed important physiological and metabolomic responses of blueberry branches to *D. eres* infection, the analyses were conducted at the tissue level and therefore could not resolve cell-type-specific defense responses. Plant immune responses are often spatially heterogeneous, with vascular and epidermal tissues exhibiting distinct transcriptional, metabolic, and redox regulation during pathogen invasion. Recent advances in single-cell RNA sequencing and spatial transcriptomics provide new opportunities to characterize these localized defense responses and should facilitate a more detailed understanding of blueberry–*D. eres* interactions [58]. In addition, this study was conducted using a single blueberry cultivar, and metabolomic profiling was limited to the early and late stages of infection, which may not fully represent cultivar-dependent responses or dynamic metabolic changes during disease development. Furthermore, pathogenicity was evaluated using detached shoots rather than whole potted seedlings. Future studies should incorporate multiple blueberry cultivars, whole-plant pathogenicity assays, and higher-resolution omics approaches. In addition, sporulation, conidial germination, melanization, and multilocus phylogenetic analyses should be included to provide a more comprehensive understanding of the biology, pathogenicity, and taxonomic status of this isolate.

## 4. Materials and Methods

### 4.1. Orchard Conditions and Experimental Materials

A survey of diseased plants was conducted at the Jilin Blueberry Orchard (42°14′ N, 127°35′ E) during the peak period of the first flush of new shoots on blueberry plants. The orchard covers an area of 1000 acres, with a soil pH of 4–6. The main varieties cultivated include ‘Reka’, ‘Polaris’, ‘Draper’ and ‘Bluegold’. Ten one-year-old ‘Bluegold’ shoots that exhibited severe disease symptoms were used for pathogen isolation. Additionally, 50 vigorous, disease-free new shoots, each 30 cm long, were selected as experimental material for pathogen back-inoculation.

### 4.2. Isolation, Purification and Identification of Pathogens

Following established methods for the isolation and purification of pathogenic fungi, appropriate modifications were implemented [59]. A 0.5 cm × 0.5 cm square sample was taken from the boundary between the diseased and healthy portions of the severely affected branch and was subsequently placed in 75% ethanol and 1% sodium hypochlorite for disinfection to eliminate surface fungi. The disinfected samples were then placed on potato dextrose agar (PDA medium) for cultivation at 25 °C under a 16/8 h dark cycle. After 3 days of incubation, peripheral mycelium was picked for further purification until a single colony formed.

The purified strain was inoculated onto fresh PDA medium, and the morphology and color of the colonies were observed during cultivation, and the morphology and structure of the spores and conidia were examined under a microscope.

In addition, ITS sequencing was performed on the purified colonies using the following primers: ITS1-F: TCCGTAGGTGAACCTGCGG, ITS4-R: TCCTCCGCTTATTGATATGC. The sequencing results were compared with sequences of strains in theNCBI database (https://www.ncbi.nlm.nih.gov/ (accessed on 11 July 2026)) and a phylogenetic tree was constructed using MEGA 12 software to preliminarily evaluate the taxonomic relationship of the isolate.

### 4.3. Pathogenicity Testing

Pathogenicity was determined according to Koch’s postulates. Approximately 50 healthy blueberry new shoots were selected and uniformly pruned into 15 cm-long stem segments. Each set of three segments constituted one treatment group, with three biological replicates. The treated stem segments were placed in conical flasks containing distilled water for in vitro culture. The shoots were scarified using a sterile scalpel to create sites for pathogen invasion. Uniformly sized fungal colonies were obtained using a sterile punch and inoculated onto the wounds on the shoots. The inoculation sites were covered with sterile moistened cotton wool to maintain high humidity. Sterile PDA medium was used as a control. The inoculated shoots were incubated at 25 °C under a 16 h light/8 h dark photoperiod. The disease symptoms of the shoots in each group were observed and recorded on the 5th and the 10th days, including lesion size and color. To fulfill Koch’s postulates, symptomatic tissues were collected from inoculated shoots after lesion development. The pathogen was re-isolated from the advancing margin of the lesions, purified on PDA medium, and identified based on morphological characteristics and ITS sequence analysis. The obtained sequences were compared with those of the original inoculated isolate to confirm pathogen identity.

### 4.4. Histochemical Staining

The cuttings were immersed completely in a staining solution containing 10 mg·mL^−1^ of 3,3′-diaminobenzidine (DAB; Solarbio, Beijing, China) and nitro blue tetrazolium (NBT; Solarbio, Beijing, China). To eliminate air bubbles, a vacuum pump (Shanghai Huxi Analytical Instrument Factory Co., Ltd., Shanghai, China) was used to evacuate the system for 30 min. Subsequently, the cuttings were stained for 24 h under light-protected conditions. Chlorophyll was entirely removed by immersing the cuttings in a decolorization solution. Photographs of the stained cuttings were taken under identical imaging conditions, and the staining intensity was analyzed using ImageJ 1.54p software. The images were converted to grayscale, and regions of the same size were selected for analysis. The relative accumulation levels of O_2_^.−^ and H_2_O_2_ were estimated by measuring the average grayscale values. Each treatment included three biological replicates.

### 4.5. Determination of O_2_^.−^ and H_2_O_2_ Content

A total of 0.3 g of the sample was ground using a grinder and mixed with phosphate-buffered solution (PBS) (pH 7.8) before being shaken thoroughly. The mixture was then centrifuged at 12,000× *g* for 10 min. The resulting supernatant was combined with PBS and hydroxylamine hydrochloride (Macklin, Shanghai, China) and incubated in a water bath at 25 °C for 30 min. Subsequently, sulfamide and α-naphthylamine (Macklin, Shanghai, China) were added, and the mixture underwent further incubation in a water bath at 25 °C for an additional 30 min. Finally, the absorbance of the solution was measured at a wavelength of 520 nm [60].

Similarly, a total of 0.3 g of the sample was ground using a grinder, mixed with PBS (pH 7.8), and shaken to achieve homogenization. The mixture was then centrifuged at 12,000× *g* for 10 min. The supernatant was combined with PBS and KI (Macklin, Shanghai, China), allowed to react in the dark for 5 min, and the absorbance of the solution was measured at a wavelength of 390 nm [60].

### 4.6. Determination of Antioxidant Enzyme Activity

A total of 0.3 g of sample was ground using a grinder, mixed with phosphate-buffered solution (PBS) (pH 7.8) and shaken to homogenize, then centrifuged at 12,000× *g* for 10 min. The supernatant was combined with a mixture of PBS, guaiacol, and 30% H_2_O_2_, incubated in the dark at 34 °C for 3 min, and POD activity was measured using a UV-visible spectrophotometer at a wavelength of 470 nm. Riboflavin (Macklin, Shanghai, China), methionine (Met; Macklin, Shanghai, China), ethylenediaminetetraacetic acid (EDTA; Macklin, Shanghai, China), nitro blue tetrazolium (NBT; Macklin, Shanghai, China), and PBS were added to the supernatant, which was then incubated in the light for 15 min, allowing for the measurement of SOD activity at a wavelength of 560 nm. Finally, PBS and distilled water were added to the supernatant, which was preheated to 25 °C before the addition of H_2_O_2_ solution, and CAT activity was measured at a wavelength of 240 nm [60].

In all antioxidant enzyme assays, a reaction mixture containing no enzyme extract was used as a blank control to correct for background levels. Enzyme activity was standardized relative to FW and expressed in units of U·g^−1^ FW. All physiological measurements were performed using three independent biological replicates, with each biological replicate analyzed in three technical replicates. 

### 4.7. Determination of MDA and REC

A total of 0.3 g of the sample was ground using a grinder, mixed with PBS (pH 7.8), and shaken to achieve homogenization. The mixture was then centrifuged at 12,000× *g* for 10 min. The supernatant was transferred to a new tube, to which thiobarbituric acid (TBA; Macklin, Shanghai, China) was added. The mixture was heated in a boiling water bath for 15 min, then cooled on ice and centrifuged at 12,000× *g* for 5 min. The MDA content was determined at wavelengths of 450 nm, 532 nm, and 600 nm [60].

A total of 5 mL distilled water was added to a tube, and the conductivity (E0) was measured using a conductivity meter. A sample weighing 0.1 g was ground and added to the tube, and the mixture was shaken for one hour before measuring the conductivity (E1). The tube was then placed in a boiling water bath for 15 min, after which the conductivity (E2) was measured. The REC was calculated using the formula: Relative conductivity = (E1 − E0)/(E2 − E0) × 100%.

### 4.8. Toluidine Blue Staining

The branches were trimmed into 4 cm sections and placed in a toluidine blue (Solarbio, Beijing, China) staining solution, followed by incubation in a 90 °C water bath for 8 h. After discarding the staining solution, the sections were immersed in a decolorization buffer (lactic acid: glycerol: anhydrous ethanol = 1:1:3) for 24 h. Subsequently, slides were prepared for examination under a microscope.

### 4.9. Metabolomics Analysis

Metabolite extraction: Approximately 25 mg of branch sample was weighed, and 1000 μL of extraction solution (including an internal standard) pre-chilled to −40 °C (methanol: acetonitrile: water = 2:2:1) was added. The sample was homogenized at 35 Hz for 4 min, sonicated in an ice-water bath for 5 min, and this process was repeated three times. Subsequently, it was allowed to stand at −40 °C for 1 h. Following this, 300 μL was transferred to a 96-well filter plate. The plate assembly was then placed in a positive-pressure device and pressurized slowly to 6 psi for 3 min. An equivalent volume of the supernatant was taken and mixed to create a QC sample.

Instrument analysis: Chromatographic separation was performed using a Phenomenex Kinetex C18 (2.1 mm × 50 mm, 2.6 μm) liquid chromatography column. The mobile phase consisted of a mobile phase A of water containing 0.01% acetic acid, and a mobile phase B of isopropanol:acetonitrile (1:1, *v*/*v*). Sample tray temperature: 4 °C, injection volume: 2 μL, and the flow rate: 0.30 mL·min^−1^. The gradient elution program was as follows: 0.00–0.50 min, 1% B; 0.50–4.00 min, linear increase from 1% to 99% B; 4.00–4.50 min, 99% B; 4.50–4.55 min, decrease from 99% to 1% B; and 4.55–6.00 min, equilibration at 1% B.

Detailed instrument parameters were as follows: Sheath gas flow rate: 50 arb, auxiliary gas flow rate: 15 arb, capillary temperature: 320 °C, full ms resolution: 60,000, MS/MS resolution: 15,000, collision energy: SNCE 20/30/40, Spray voltage: 3.8 kV (positive) or −3.4 kV (negative).

Data processing: Raw data were converted using ProteoWizard software (V3.0.24054) for metabolite identification and visualization analysis, with reference to the in-house database (standard library) and BT-Plant (V1.1, plant-specific library).

Before statistical analysis, peak extraction, alignment and standardization were performed on the original metabolomics data. Metabolite abundance was standardized based on total ion current to minimize systematic variation between samples. Metabolites with too many missing values or low repeatability were eliminated before subsequent analysis.

QC samples were prepared by pooling all samples in equal proportions and were analyzed throughout the entire process to assess instrument stability and data reproducibility. Supervised orthogonal partial least squares discriminant analysis (OPLS-DA) was employed to further evaluate metabolic differences between treatment groups. The robustness and predictive ability of the OPLS-DA model were assessed by permutation test to evaluate model validity and avoid overfitting. Differential accumulation metabolites were selected based on the following criteria: variable importance in projection (VIP) ≥ 1, absolute log_2_ fold change (|log_2_FC|) ≥ 1, and *p*-value < 0.05.

Metabolomic analysis was primarily conducted using an LC-MS/MS system equipped with a quadrupole-Orbitrap mass spectrometer (Thermo Fisher Scientific, Waltham, MA, USA). Univariate and multivariate statistical analyses (MVA) were performed on the qualitative and quantitative results of the metabolome to identify significantly differentially expressed metabolites. Building upon the basic data analysis, a series of bioinformatics analyses were conducted to visualize the differentially expressed metabolites and explore their biological functions.

### 4.10. Data Analysis

All experiments included at least three biological replicates, with three technical replicates performed for each biological replicate. Prior to conducting one-way analysis of variance (ANOVA), the Shapiro–Wilk test and Levene’s test were used to assess the normality of the data and the homogeneity of variances, respectively. Statistical analysis was performed using SPSS 26.0 software (IBM, Armonk, NY, USA); to determine statistical significance, one-way ANOVA followed by Tukey’s honestly significant difference (HSD) test was performed to determine the significance of differences among treatments (*p* < 0.05).

## 5. Conclusions

Based on morphological characteristics and ITS sequence analysis, this study preliminarily identified the pathogen associated with blueberry canker in Jilin as *D. eres*. Physiological analysis showed that pathogen infection induced significant ROS accumulation in blueberry shoots and activated the antioxidant defense system. Metabolomic analysis further revealed extensive metabolic reprogramming following pathogen infection, especially in metabolic pathways such as phenylpropanoid biosynthesis and flavonoid biosynthesis. After infection, a variety of flavonoids and phenolic acid-related metabolites, including quercetin, kaempferol, and ferulic acid, were significantly accumulated, indicating that these metabolites may be related to the defense response of blueberry against pathogen-induced oxidative stress.

Integrated physiological and metabolomic analyses suggested that pathogen-induced oxidative stress was accompanied by coordinated activation of antioxidant defenses and secondary metabolism, which may contribute to defense responses in blueberry shoots. These findings provide new insights into blueberry–*Diaporthe* interactions and the metabolic basis of blueberry defense responses. Nevertheless, the specific functions of these defense-associated metabolites and the taxonomic identity of the isolate require further confirmation through functional experiments and multilocus phylogenetic analyses. Based on the infection characteristics of *D. eres* and the observed activation of defense-related metabolism, orchard management practices such as reducing excessive humidity, improving canopy ventilation, removing infected branches, and maintaining balanced plant nutrition may contribute to disease suppression, although these strategies require further field evaluation.

## Data Availability

The original contributions presented in this study are included in this article. Further inquiries can be directed to the corresponding authors.

## Supplementary Materials

The following supporting information can be downloaded at: https://www.mdpi.com/article/10.3390/plants15142172/s1, Figure S1. Assessment of colony morphology and pathogenicity in several purified fungal strains. Figure S2. Trypan blue staining of blueberry new shoots infected with *Diaporthe eres*. Figure S3. Scatter plot of the PCA model comparing Group CK-0 with Group CK-10 and Group D-0 with Group D-10. Figure S4. OPLS-DA analysis and permutation tests of metabolomic profiles during *D. eres* infection. Figure S5. Hierarchical cluster analysis heatmap comparing CK-0 and D-10. Figure S6. Hierarchical cluster analysis heatmap comparing CK-10 and D-10. Figure S7. Hierarchical cluster analysis heatmap comparing D-0 and D-10.
