# Supplementary material for: Physiological and Metabolomic Responses of ‘Bluegold’ Blueberry to Infection by an Isolate Preliminarily Identified as Diaporthe eres
Source: Plants (Basel). 2026 Jul 15;15(14):2172. doi: 10.3390/plants15142172 (PMC13414685; doi:10.3390/plants15142172)
Supplement: Supplementary file 1 [file plants-15-02172-s001.zip › plants-4423946-supplementary.pdf]

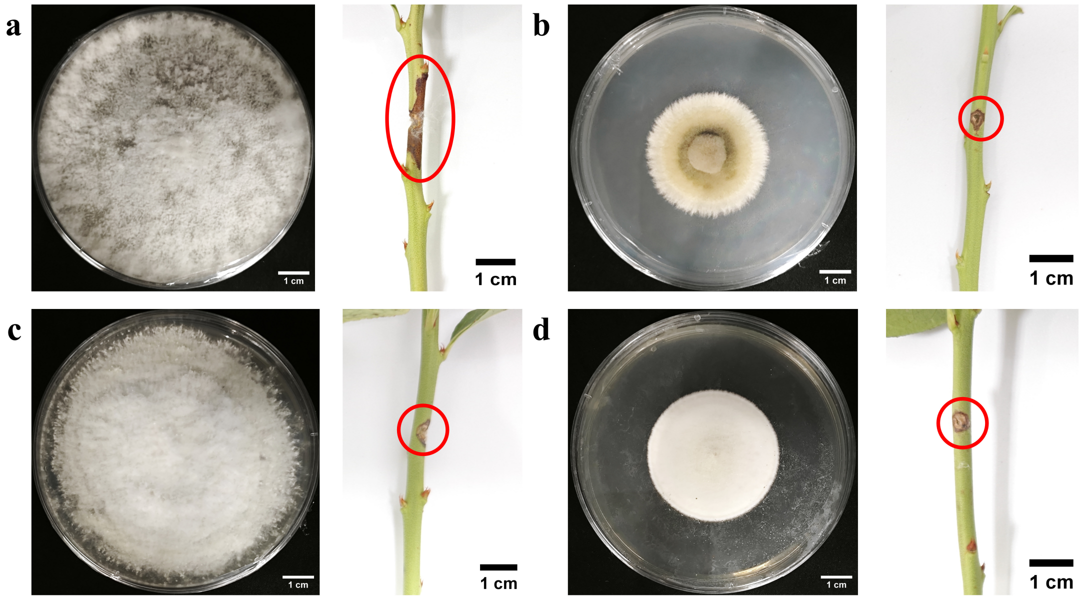

Figure S1. Assessment of colony morphology and pathogenicity in several purified fungal strains. (a)*Diaporthe*; (b) *Epicoccum*; (c) *Acremonium*; (d) *Simplicillium*.

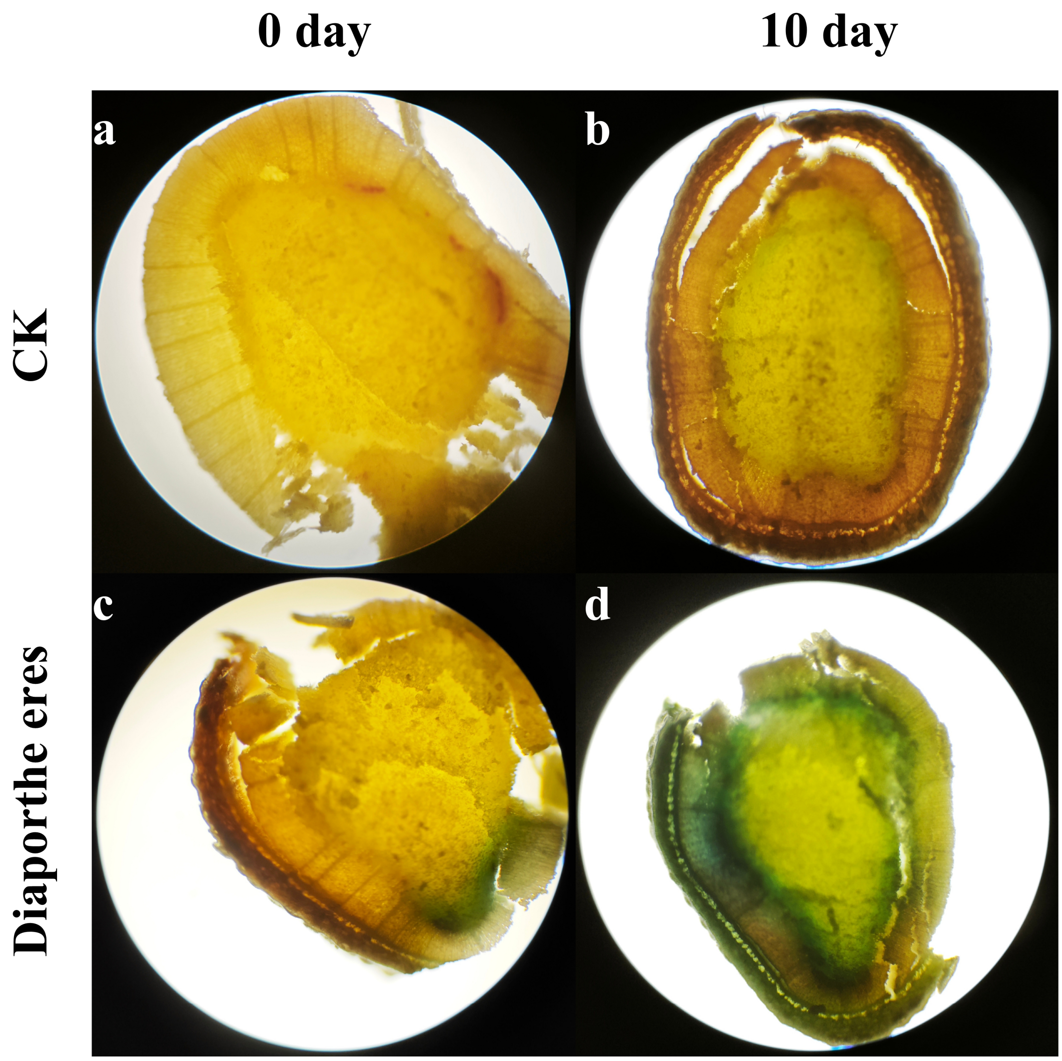

Figure S2. Trypan blue staining of blueberry new shoots infected with *Diaporthe eres*. (a,b) Cross-sections of blueberry branches without infection and (c,d) with infection by pathogenic bacteria at 0 day and 10 days.

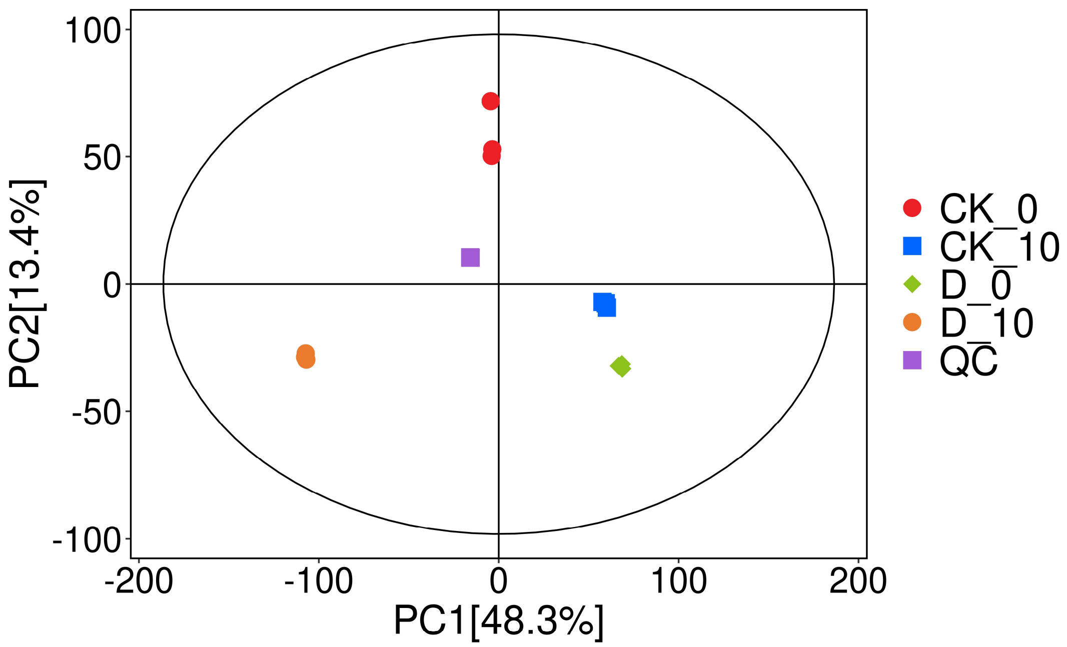

Figure S3. Scatter plot of the PCA model comparing Group CK-0 with Group CK-10 and Group D-0 with Group D-10. The x-axis (PC1) and y-axis (PC2) represent the scores for the first and second principal components, respectively. Each scatter plot represents a sample, with the colour and shape of the scatter plots indicating different groups.

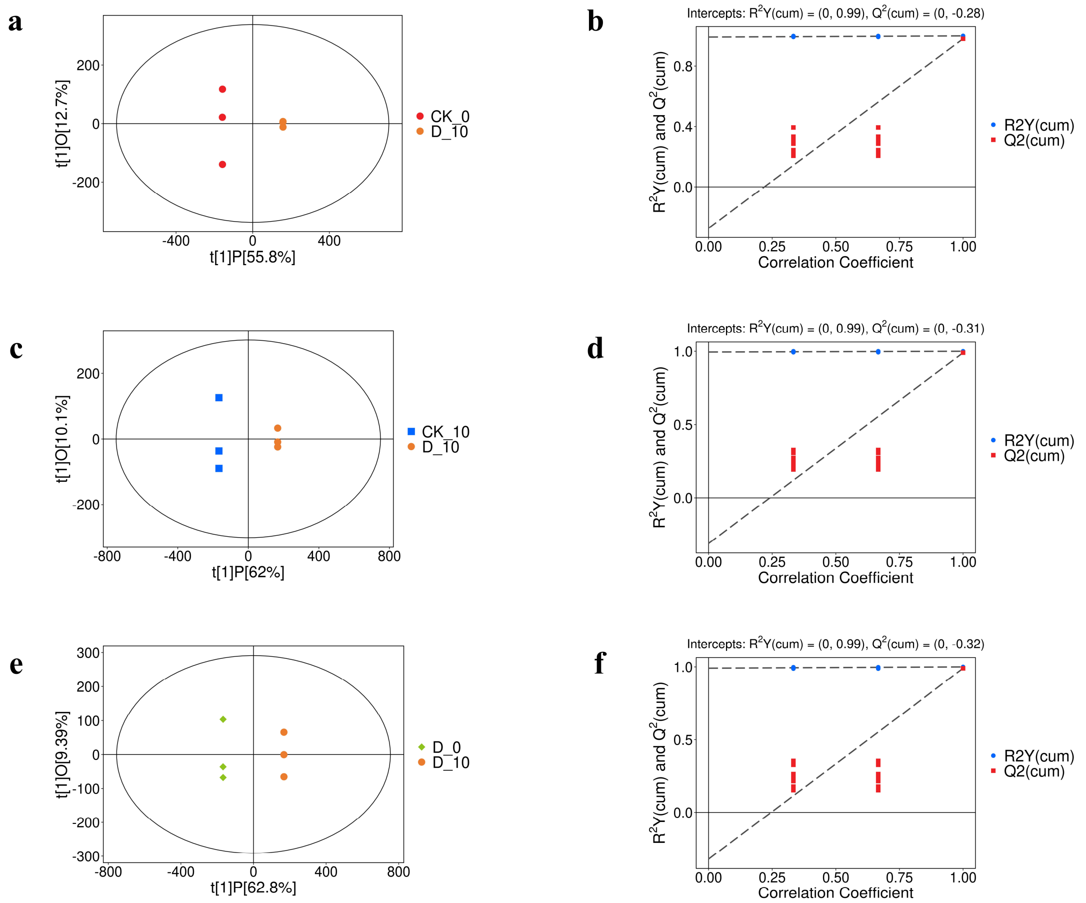

Figure S4. OPLS-DA analysis and permutation tests of metabolomic profiles during D. eres infection. (a) OPLS-DA Analysis of CK-0 vs D-10; (b) permutation test for CK-0 vs D-10; (c) OPLS-DA Analysis of CK-10 vs D-10; (d) permutation test for CK-10 vs D-10; (e) OPLS-DA Analysis of D-0 vs D-10; (f) permutation test for D-0 vs D-10.

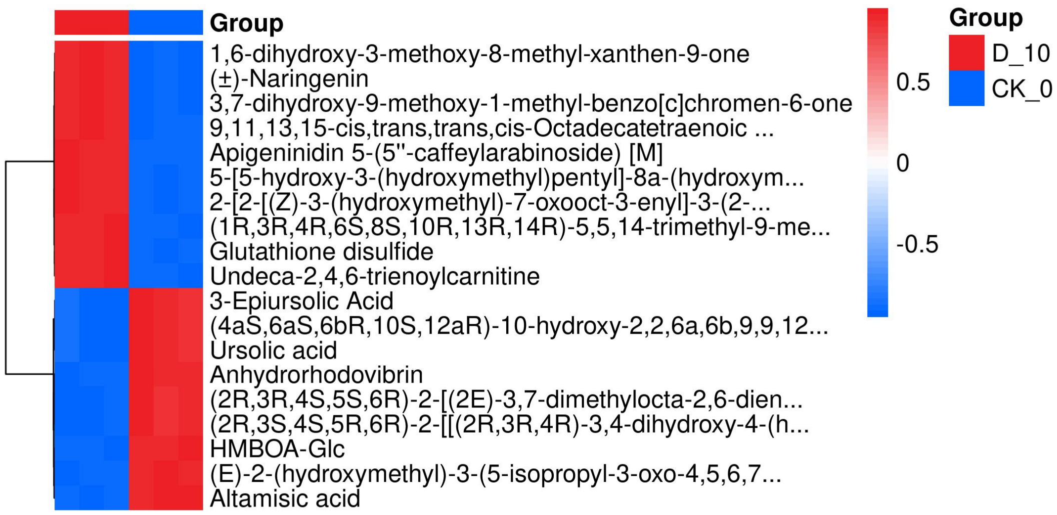

Figure S5. Hierarchical cluster analysis heatmap comparing CK-0 and D-10(p-value < 0.01).

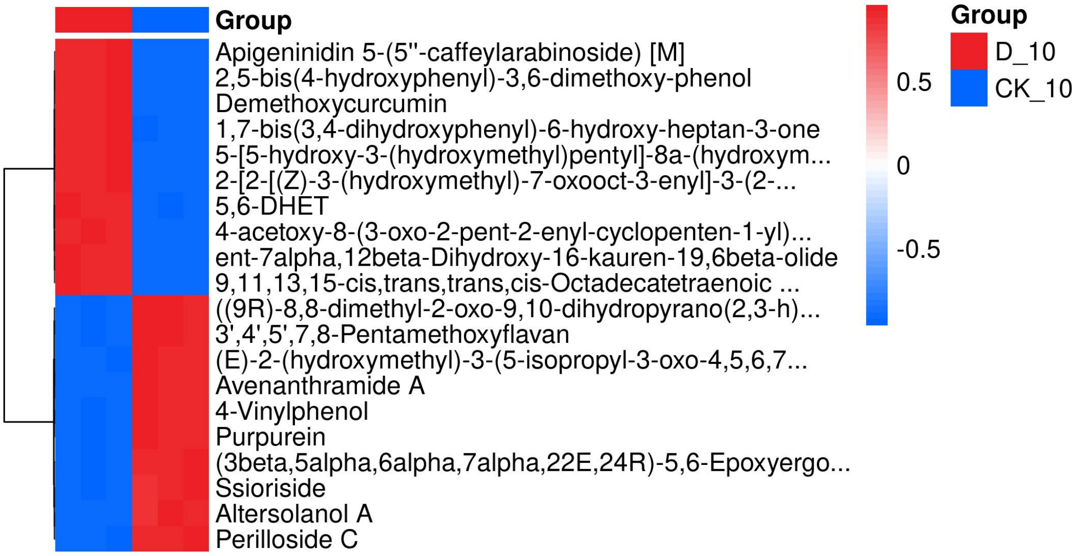

Figure S6. Hierarchical cluster analysis heatmap comparing CK-10 and D-10(p-value < 0.01).

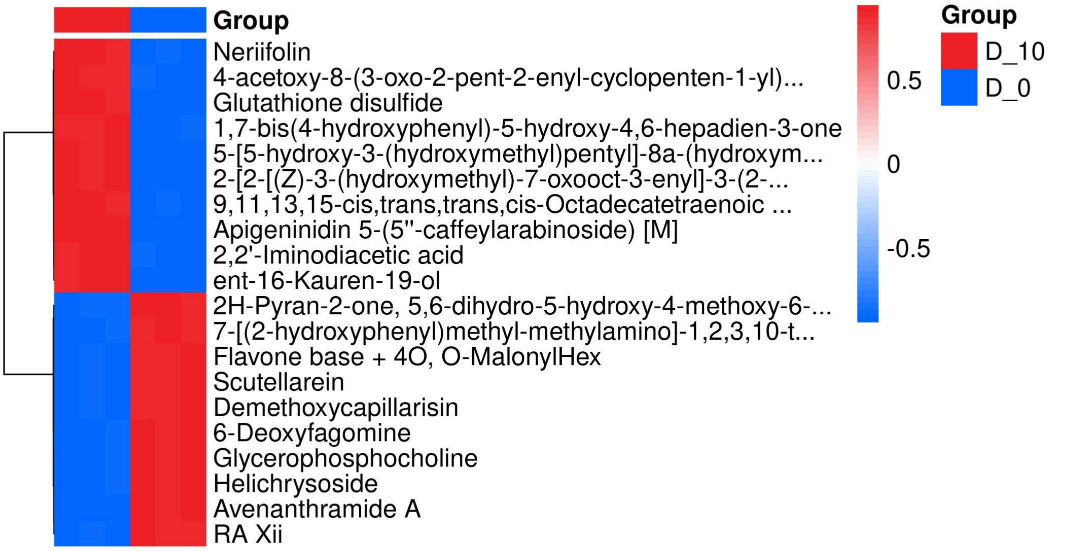

Figure S7. Hierarchical cluster analysis heatmap comparing D-0 and D-10(p-value < 0.01).
